# Inferior Pancreaticoduodenal Artery Pseudoaneurysm Causing Biliary Obstruction: A Case Report

**DOI:** 10.5811/cpcem.1598

**Published:** 2024-03-26

**Authors:** Patrick Meloy, Will S. Lindquester, Jeffrey Stebbins, Elaine Bromberek

**Affiliations:** * Emory University School of Medicine, Department of Emergency Medicine, Atlanta, Georgia; † Emory University School of Medicine, Division of Interventional Radiology and Image-guided Medicine, Atlanta, Georgia

**Keywords:** *case report*, *visceral pseudoaneurysm*, *pancreaticoduodenal pseudoaneurysm*, *obstructive jaundice*, *alcohol use disorder*

## Abstract

**Introduction:**

Visceral arterial aneurysms and pseudoaneurysms are rare but dangerous pathologies, with reported incidence of 0.01–0.2% of the worldwide population, as found on autopsy. Pancreaticoduodenal artery pathology accounts for approximately 2% of all visceral aneurysms; it is commonly caused by chronic inflammatory processes, such as pancreatitis or adjacent pseudocysts. Morbidity and mortality commonly result from rupture of the aneurysm itself, leading to life-threatening hemorrhage into the peritoneum or gastrointestinal tract.

**Case Report:**

Here we present the case of a 64-year-old male patient with previous history of alcohol use disorder leading to chronic pancreatitis and prior embolization of an inferior pancreaticoduodenal pseudoaneurysm, who presented to the emergency department (ED) with abdominal pain, nausea, and vomiting, and was found to have a large recurrent inferior pancreaticoduodenal pseudoaneurysm with associated obstructive cholangitis and pancreatitis via contrast-enhanced computed tomography (CT) of the abdomen and pelvis. The patient was managed emergently by interventional radiology angiography with embolic coiling and percutaneous biliary catheter placement, and he subsequently underwent biliary duct stenting with gastroenterology. The patient was successfully discharged after a brief hospitalization after resolution of his pancreatitis and associated hyperbilirubinemia.

**Conclusion:**

Pancreaticoduodenal artery aneurysms and pseudoaneurysms are rare and dangerous visceral pathologies. Patients can be diagnosed rapidly in the ED with CT imaging and need urgent endovascular management to prevent morbidity and mortality.

Population Health Research Capsule What do we already know about this issue?

*Visceral artery aneurysms are rare but dangerous. Pancreatitis is a cause of pancreaticoduodenal artery aneurysm, and rupture can lead to hemorrhage into the peritoneum.*

What was the research question?

*This case details an aneurysm present in up to 0.2% of the worldwide population. Its size and location led to biliary obstruction, which aided in rapid diagnosis.*

What was the major finding of the study?

*Visceral artery aneurysms are diagnosed with CT angiography; consideration should be given to urgent interventional radiology coiling or embolization for stability.*

How does this improve population health?

*Clinicians should consider visceral artery aneurysms in patients with unexplained obstructive biliary pathology and obtain CT angiography for diagnosis.*

## INTRODUCTION

Visceral artery aneurysms are clinically rare entities and are typically found incidentally on abdominal imaging or via autopsy.^1^^,^^2^ Patients who present with symptoms, such as abdominal pain, vomiting, or gastrointestinal bleeding, are more likely to be experiencing a true emergency, with 8.5% of all cases resulting in death.^2^ Aneurysms of the pancreaticoduodenal arteries represent 2% of all visceral aneurysms and are the most life-threatening.^2^ Compared to true aneurysms, patients with pseudoaneurysms have profoundly higher rupture rates, up to 76% compared to 3%, and require emergent treatment for stabilization.^3^

In this case, the patient presented with symptomatic abdominal pain and tenderness and was found to have a very large recurrent pseudoaneurysm of the inferior pancreaticoduodenal artery, the size of which led to obstructive biliary disease and cholangitis. The patient underwent emergent embolization of his pseudoaneurysm and percutaneous biliary catheter placement with interventional radiology (IR) to manage his severe disease process.

## CASE REPORT

A 64-year-old male patient with a past medical history of pancreatitis, alcohol use disorder, glaucoma, and prior gastrointestinal bleed, presented to the emergency department (ED) complaining of two weeks of abdominal pain, nausea, and vomiting. He reported that he had chronic abdominal pain that had worsened in the prior two weeks. He was hospitalized at an outside facility one month prior for a gastrointestinal bleed, but a source of bleeding was never identified. His surgical history was significant for coil embolization of an inferior pancreaticoduodenal artery (IPDA) pseudoaneurysm sac, measuring 2.2 centimeters (cm) at the time of embolization, with additional coiling of the gastroduodenal artery (GDA) to prevent collateral filling of the pseudoaneurysm. He had also undergone total knee replacement.

On arrival, his vital signs were temperature 36.8° Celsius (C) (oral), heart rate 85 beats per minute, respiratory rate 16 breaths per minute, blood pressure 139/67 millimeters of mercury, and oxygen saturation 99% on room air. His physical exam was notable for scleral icterus and moderate abdominal tenderness, worse over the epigastrium, but without rebound tenderness or guarding. Intravenous (IV) access was established, labs were drawn, and the patient was given one liter lactated Ringer’s, 4 milligrams (mg) IV morphine, and 4 mg IV ondansetron.

The patient’s laboratory studies were significant for an initial white blood cell count (WBC) of 14.0 per microliter (10^9^/liter) (reference range 4.5–11.0 × 10^9^/liter), hemoglobin 8.6 grams per deciliter (g/dL) (14–18 g/dL), hematocrit 26.6% (41–50%), and platelets of 486 × 10^9^/liter (150–400 × 10^9^/liter). Lipase was elevated at 225 units per liter (U/L) (0–160 U/L). Liver function tests were also obtained and were concerning for total bilirubin 6.0 mg/dL (0.1–1.2 mg/dL), aspartate transaminase 102 U/L (8–33 U/L), and alanine transaminase 127 U/L (7–56 U/L). The patient had evidence of coagulopathy with prothrombin time of 21 seconds (10–13 seconds) and international normalized ratio 1.86 (reference range less than 1.1), despite lack of any systemic anticoagulation.

**Image 1.**
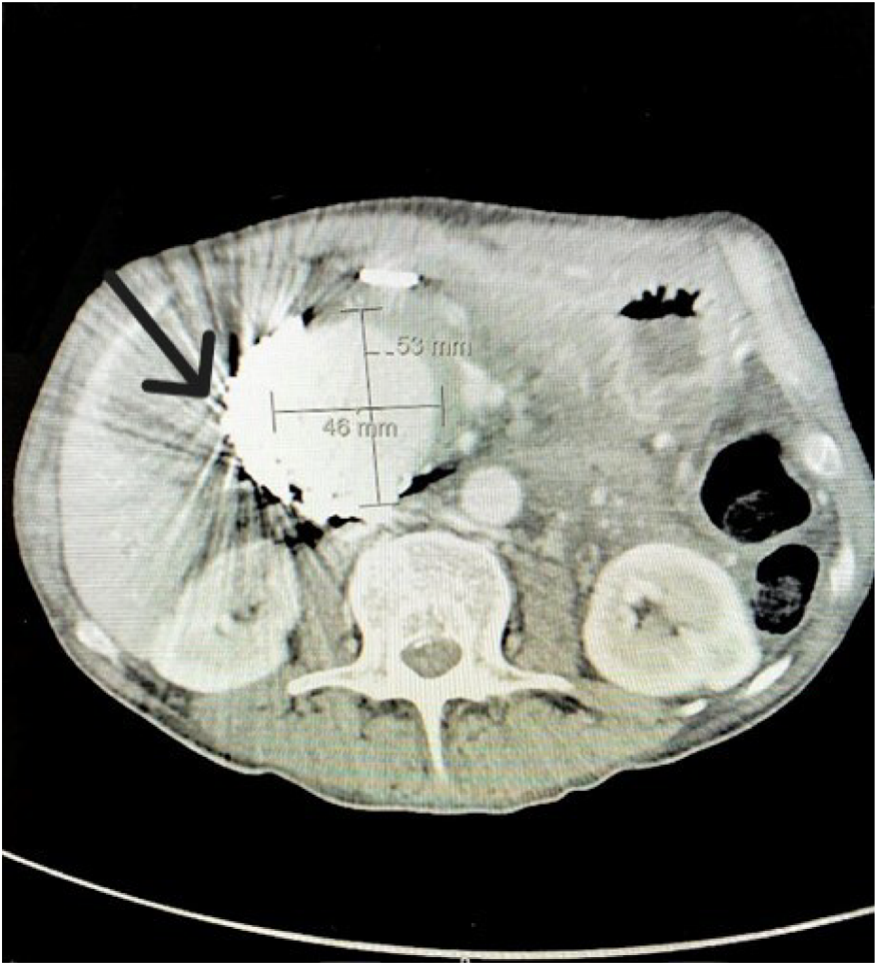
Axial contrast-enhanced computed tomography in the arterial phase, pre-procedure, demonstrating a large visceral pseudoaneurysm in the right upper quadrant (brackets with measurements); and an artifact from the endovascular coils within the lesion from prior embolization (arrow).

**Image 2.**
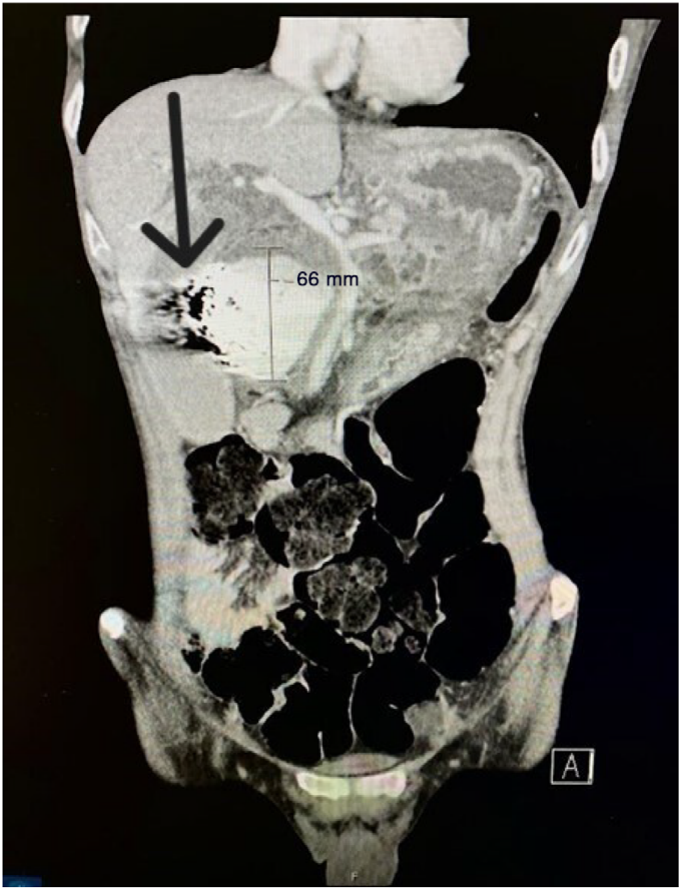
Coronal contrast-enhanced computed tomography in the arterial phase, pre-procedure, demonstrating a large visceral pseudoaneurysm in the right upper quadrant (brackets with measurements); and an artifact from the endovascular coils within the lesion from prior embolization (arrow).

**Image 3.**
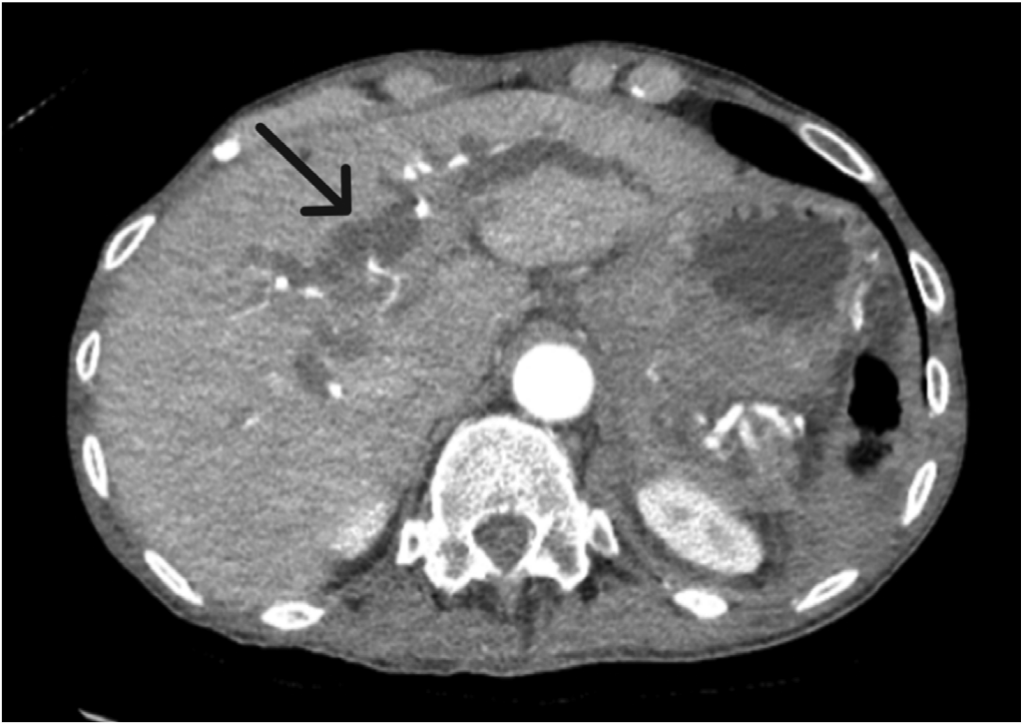
Axial contrast-enhanced computed tomography in the arterial phase, pre-procedure, demonstrating severe intra-hepatic biliary ductal dilation (arrow).

Initial computed tomography (CT) with IV contrast of the abdomen and pelvis revealed a 6.6-cm enhancing lesion in the region of the pancreatic head and common bile duct, consistent with a large visceral pseudoaneurysm without arterial extravasation (Images 1 and 2). Coils from the prior embolization were present within the pseudoaneurysm sac, indicating that this was a recurrence with significant enlargement of the previously treated pseudoaneurysm. Severe intrahepatic biliary ductal dilation and diffuse dilation of the gallbladder was also seen, with the presumed GDA pseudoaneurysm causing mass effect on the common bile duct (Image 3).

The imaging results were discussed immediately with IR, and the patient was prepped for emergent embolization. Additionally, IR planned urgent percutaneous biliary catheter placement given concern for cholangitis in the setting of an elevated WBC count and obstructive cholangiopathy. Angiography demonstrated a large IPDA pseudoaneurysm arising from the branches of the superior mesenteric artery. Coil embolization of the arterial inflow and outflow was successful with no persistent filling of the pseudoaneurysm post embolization, with associated preservation of the surrounding jejunal arteries via collaterals.

Given that the patient was also found to have biliary ductal dilation with obstruction and concern for cholangitis, a left-sided biliary drain was placed for decompression. The patient tolerated the initial procedure and was admitted to the surgical intensive care unit for ongoing monitoring. Over the following four days, he had improvement of his liver function tests and leukocytosis and was subsequently discharged home. Five weeks after discharge, the patient had repeat imaging of the abdomen and pelvis, which did not show any patent pseudoaneurysm present. He subsequently underwent biliary stent placement with gastroenterology and removal of his biliary catheter.

## DISCUSSION

A pseudoaneurysm is defined as an encapsulated hematoma in communication with the lumen of the ruptured vessel, where the external wall consists of adventitia, perivascular tissue, fibrosis, or clot.^2^ Pseudoaneurysms usually occur in the proximity of pseudocysts, which erode into and communicate with a vessel to create a pseudoaneurysm.^2^^,^^3^ Pseudoaneurysms usually do not occur immediately after an episode of acute pancreatitis but are more commonly found 3–5 weeks after the initial episode. Bleeding and hemorrhage have been seen anywhere from two months to eight years after a single episode of pancreatitis.^5^^,^^6^ Gastroduodenal artery pseudoaneurysms are seen in up to 20% of arterial pseudoaneurysms complicated by pancreatitis, while pancreaticoduodenal arteries are involved in up to 10% of cases.^2^^,^^5^

Although pancreatic pseudoaneurysms are uncommon, it is important to recognize this condition early, as it can result in life-threatening complications. Patients will typically present with gastrointestinal bleeding or abdominal pain.^2^ Pseudoaneurysms may cause gastrointestinal bleeding by erosion into the adjacent bowel or they may directly rupture, causing bleeding into the retroperitoneum.^1^ Computed tomography usually provides appropriate diagnostic images; however, angiography has been demonstrated to be the most informative investigation for diagnosis as well as treatment. Computed tomography angiography has a high rate of sensitivity and specificity but does not facilitate intervention concurrently. Angiography defines the character and location of the lesions, as well as provides an opportunity to gain control over the bleeding by transcatheter embolization or possible stenting.^1^ A series of 35 patients from 1993–2003 indicated 95% of pseudoaneurysms were detected with angiography while only 90% were detected with CT angiography.^1^ Although CT angiography is an important tool in the ED to diagnose these cases, management per the IR team is paramount for success.

Endovascular use of metallic coils is frequently used as the definitive treatment of these pseudoaneurysms. Other tools including covered stents, detachable balloons, gel foam, or particles have also been used with success rates of up to 85%.^1^ Surgical treatment has been shown to be challenging and associated with high morbidity rates, thus reserving surgical intervention in cases of failed embolization or hemodynamically unstable patients.^1^ The reported incidence of recurrent hemorrhage after thrombosis is as high as 30%, with embolization of pancreatic pseudoaneurysms requiring long-term follow-up in these patients.^13^ In the case of our patient, IR was able to successfully embolize the large IPDA pseudoaneurysm with follow-up imaging demonstrating ongoing resolution.

## CONCLUSION

Pancreaticoduodenal and gastroduodenal artery pseudoaneurysms are rare and dangerous visceral pathologies. Pseudoaneurysms usually occur in the proximity of pseudocysts or chronic inflammatory conditions and are a known complication of chronic pancreatitis. Patients can be diagnosed in the ED with CT angiography and need urgent endovascular management with interventional radiology to prevent morbidity and mortality. Due to high rates of recurrent hemorrhage after thrombosis, patients should have close follow-up and serial imaging to assess for pseudoaneurysm or aneurysm recurrence.
